# Smad Phospho-Isoforms for Hepatocellular Carcinoma Risk Assessment in Patients with Nonalcoholic Steatohepatitis

**DOI:** 10.3390/cancers12020286

**Published:** 2020-01-24

**Authors:** Kanehiko Suwa, Takashi Yamaguchi, Katsunori Yoshida, Miki Murata, Mayuko Ichimura, Koichi Tsuneyama, Toshihito Seki, Kazuichi Okazaki

**Affiliations:** 1 Department of Gastroenterology and Hepatology, Kansai Medical University 2-5-1, Shin-Machi, Hirakata, Osaka 573-1010, Japan; suwakan@hirakata.kmu.ac.jp (K.S.); yoshidka@hirakata.kmu.ac.jp (K.Y.); muratami@takii.kmu.ac.jp (M.M.); sekit@mc.kmu.ac.jp (T.S.); okazaki@hirakata.kmu.ac.jp (K.O.); 2 Department of Pathology & Laboratory Medicine, Institute of Biomedical Sciences, Tokushima University Graduate School. 3-18-15 Kuramoto, Tokushima 770-8503, Japan; ichimura.mayuko@tokushima-u.ac.jp (M.I.); tsuneyama.koichi@tokushima-u.ac.jp (K.T.)

**Keywords:** non-alcoholic steatohepatitis, Smad, hepatocellular carcinoma

## Abstract

Nonalcoholic steatohepatitis (NASH)-related hepatocellular carcinoma (HCC) sometimes occurs in mildly fibrotic livers, while HCC incidence in NASH-related cirrhosis is lower than and less predictable than in hepatitis C virus (HCV)-related cirrhosis. Transforming growth factor (TGF)-β signaling in hepatocytic nuclei is implicated in fibrosis and carcinogenesis. TGF-βtype I receptor (TβRI) and c-Jun N-terminal kinase (JNK) differentially phosphorylate the mediator Smad3, resulting in 2 distinct phospho-isoforms: C-terminally phosphorylated Smad3 (pSmad3C) and linker-phosphorylated Smad3 (pSmad3L). In mature hepatocytes, oncogenic signaling via the JNK/pSmad3L pathway antagonizes signaling via the tumor-suppressive TβRI/pSmad3C pathway. We immunohistochemically examined domain-specific Smad3 phosphorylation in liver biopsy specimens from 30 NASH patients representing different fibrotic stages and 20 chronically infected hepatitis C patients as controls, correlating Smad3 phosphorylation with clinical course. HCC occurred during follow-up in 11 of 12 NASH patients with abundant pSmad3L and limited pSmad3C but in only 2 of 18 with limited pSmad3L. In contrast, HCC developed in 12 of 15 NASH patients with limited pSmad3C but only 1 of 15 with abundant pSmad3C. Two of fourteen NASH patients with mild fibrosis developed HCC, their hepatocytic nuclei showed abundant pSmad3L and limited pSmad3C. Five of sixteen patients with severe fibrosis did not develop HCC, their hepatocytic nuclei showed limited pSmad3L and abundant pSmad3C. Smad phospho-isoforms may represent important biomarkers predicting HCC in NASH and potential therapeutic targets for preventing NASH-related HCC.

## 1. Introduction

Hepatocellular carcinoma (HCC) ranks third among causes of cancer-related death worldwide [1]. Chronic infections with hepatitis B virus (HBV) and hepatitis C virus (HCV) represent the most significant causes of HCC [2]. As recent advances in anti-HBV and HCV therapies have reduced HCC occurrence, HCC development in nonalcoholic fatty liver disease (NAFLD) is increasing in importance [3]. Rising obesity rates over the last 20 years have increased prevalence of metabolic syndrome [4,5,6], a cause of NAFLD including a progressive inflammatory form, nonalcoholic steatohepatitis (NASH) [7,8]. In Japan, HCV-related HCCs account for 67% of all HCCs, followed by HCCs related to HBV infection at 16%, while 15.8% of HCC patients have non-viral liver disease [9]. Among non-viral chronic liver diseases, natural histories of autoimmune hepatitis, primary biliary cholangitis, and alcoholic liver disease are better understood than that of NAFLD. In the first 3 diseases, HCC typically arises through cirrhosis complicating prolonged chronic hepatic inflammation. Unfortunately, cirrhosis develops in over 80% of those patients [10]. In NAFLD, however, cirrhosis is less frequent, platelet counts (PLT) also are higher. Among 258 NAFLD patients with compensated cirrhosis, followed up for a median of approximately 2 years, Synyal et al. reported that only 5 (<2%) developed HCC [11]. PLT, serum concentrations of type IV collagen 7 s domain and of Wisteria floribunda agglutinin-positive Mac-2 binding protein, the fibrosis-4 (FIB-4) index, and liver rigidly determined by imaging technologies represent relatively noninvasive surrogate measurements for estimating degree of liver fibrosis in patients with NAFLD [12,13,14,15,16]. Furthermore, NASH-related HCC sometimes occurs in mildly fibrotic livers [10,17]. Accurately defining the population at high risk for HCC in NASH, therefore, would be difficult.

**Figure 1:**
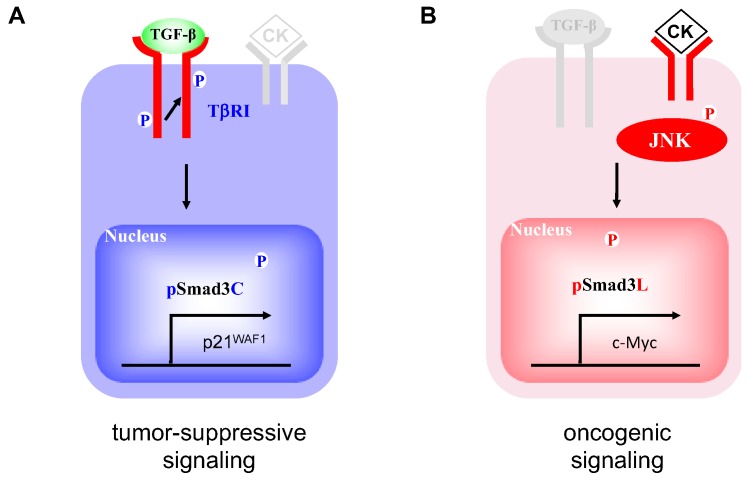
Smad3-dependent signaling between two distinct phospho-isoform. (**A**) Catalytically active TβRI phosphorylates C-terminal region of Smad3. pSmad3C interferes with cell-cycle progression by transcriptional activation of p21^waf1^ in the nucleus. (**B**) Pro-inflammatory cytokines (CK) activate c-Jun N-terminal kinase (JNK), resulting in phosphorylation of Smad3 at linker domain. pSmad3L promotes hepatocyte proliferation and hepatic carcinogenesis by up-regulating c-Myc transcription in the nucleus.

Transforming growth factor (TGF)-β, an important cytokine regulating the hepatic cell cycle, cell differentiation, and synthesis of extracellular matrix (ECM) components, is involved in many pathologic processes including fibrosis and carcinogenesis [18,19,20,21]. Smads, which consist of MH1, MH2, and linker domains, are central mediators of signals from the receptors for TGF-β superfamily members to the nucleus [22]. TGF-β binds to type II receptors on the cell surface and activates type I receptors (TβRI). Activated TβRI phosphorylates the C-terminal region of receptor-activated Smads, which include Smad2 and a closely related protein, Smad3. Linker domains of Smad2/3 are phosphorylated by Ras-related kinases including c-Jun N-terminal kinase (JNK) [23]. TβRI and JNK differentially phosphorylate Smad3 to result in 2 distinct phospho-isoforms: C-terminally phosphorylated Smad3 (pSmad3C) and linker-phosphorylated Smad3 (pSmad3L) [24,25]. TGF-β-dependent pSmad3C signaling interferes with cell-cycle progression by transcriptional activation of p15^INK4B^ and p21^CIP1^ and repression of c-Myc genes [26,27,28]. In this manner, pSmad3C carries out cytostatic/tumor-suppressive TGF-β signaling (Figure 1A) [29,30,31]. On the other hand, JNK-mediated pSmad3L promotes hepatocyte proliferation and hepatic carcinogenesis by up-regulating c-Myc transcription (Figure 1B) [32]. Most importantly, the pSmad3L-mediated proliferative effect antagonizes cytostatic pSmad3C signaling [24]. A key therapeutic aim in preventing or arresting hepatic carcinogenesis is restoration of the lost tumor-suppressive function observed in normal hepatocytes. This difficult aim has been accomplished in several studies. Administered to rats the JNK inhibitor SP600125 suppresses chemically induced hepatocarcinogenesis by shifting phosphorylation from the oncogenic pSmad3L signaling to the tumor-suppressive pSmad3C pathway [33]. In livers of HCV or HBV infected patients, chronic inflammation and hepatitis virus components cooperatively shift Smad signaling from the tumor-suppressive pSmad3C pathway to the carcinogenic pSmad3L pathway, accelerating liver fibrosis and increasing risk of HCC [34,35]. Effective antiviral treatment can reverse Smad phospho-isoform signaling from the oncogenic pSmad3L pathway to the tumor-suppressive pSmad3C pathway [36,37]. These observations suggest that the TGF-β-mediated Smad signaling is an important target for therapies aiming to reduce emergence of HCC in chronic liver disorders.

Our present studies extend the previous observations to NASH-related carcinogenesis. We investigated Smad phospho-isoform signaling at each stage of fibrosis in NASH and its association with HCC occurrence. During progression of NASH-related liver disease, pSmad3L pathway in hepatocytes tended to increase, on the other hand, pSmad3C pathway decreased. Interestingly, NASH patients whose livers show pSmad3L dominance in hepatocytic nuclei can develop HCC in the near future even when relatively little fibrosis is present. Alternatively, pSmad3C dominance opposes HCC development even in cirrhotic livers. These data suggest that Smad phospho-isoforms may be important biomarkers for prediction of HCC in NASH, and also may represent a potentially therapeutic target for prevention of NASH-related HCC.

## 2. Results

### 2.1. Two Distinct Hepatocytic Smad Phospho-Isoform Signaling Pathways in NASH Livers: pSmad3L- and pSmad3C-Dominant Types

To investigate domain-specific phosphorylation mediating Smad3 signaling in vivo, we generated Abs specific to each phosphorylation site [38]. We performed immunohistochemistory using domain-specific phospho-Smad3 Abs in liver biopsy specimens from 30 NASH patients representing different fibrotic stages and 20 chronically infected hepatitis C patients as controls. We semiquantitatively scored hepatocytic Smad3 phosphorylation from 0 to 4 as described in Methods and investigated the correlation between phosphorylation score and disease progression [34]. Table 1 and Table 2 show clinical backgrounds and positivity scores for pSmad3L and pSmad3C in liver specimens from 30 patients with NASH and 20 from patients with HCV-related chronic liver diseases, respectively. Distribution of pSmad3L and pSmad3C in NASH specimens showed 2 distinct patterns at various severities of fibrosis.

**Figure 2:**
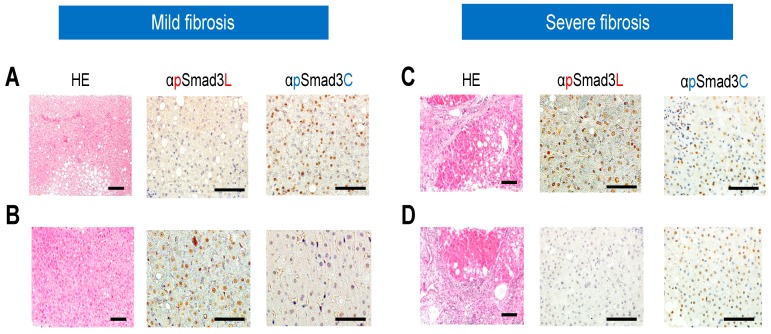
Two distinct hepatocytic Smad phospho-isoform signaling pathways in livers with nonalcoholic steatohepatitis (NASH): pSmad3L-dominant and pSmad3C-dominant. (**A**) This liver specimen showing mild fibrosis (hematoxylin-eosin on HE column) was obtained from patient 5 in Table 1, who had not developed hepatocellular carcinoma (HCC) for 15 years following histopathologic diagnosis of NASH. Hepatocytes retained phosphorylation at Smad3C (α pSmad3C column) but showed little phosphorylation at Smad3L (α pSmad3L column). (**B**) This liver specimen was obtained from patient 18 in Table 1, who developed HCC within 8 years after liver biopsy. The specimen showed degrees of fibrosis and necroinflammatory activity similar to those in (**A**). Smad3 in hepatocytic nuclei was phosphorylated sparsely in the C-terminal region (α pSmad3C column) but intensely in the linker region (α pSmad3L column). (**C**) In the cirrhotic liver specimen obtained from patient 26 in Table 1 who was diagnosed with HCC at the time of liver biopsy, phosphorylation of Smad3L in hepatocytic nuclei was high, while C-terminal phosphorylation of Smad3 was suppressed. (**D**) In another cirrhotic liver specimen, obtained from patient 17 in Table 1, HCC had not developed during 13 years following liver biopsy. Hepatocytic nuclei showed high phosphorylation at Smad3C but low phosphorylation at Smad3L. The specimen showed degrees of fibrosis and necroinflammatory activity similar to those in (**C**). Formalin-fixed, paraffin-embedded liver sections were stained with anti-pSmad3L Ab (αpSmad3L column) and anti-pSmad3C Ab (αpSmad3C column). Abs then were bound by goat anti-rabbit IgG conjugated with peroxidase-labeled polymer. Peroxidase activity was detected using 3,3′-diaminobenzidine tetrahydrochloride. Antibody-treated sections were counterstained with hematoxylin (blue). Brown color indicates specific Ab reactivity.

In a mildly fibrotic liver specimen with necroinflammatory grade 1 and fibrosis stage 1 from patient 5 in Table 1, who has not developed HCC during 15 years of follow-up, many hepatocytes retained phosphorylation at Smad3C, while phosphorylation at Smad3L was scant (Figure 2A). Another liver specimen with a similar necroinflammatory grade and a mild fibrosis stage was obtained from patient 18 in Table 1, who developed HCC within 8 years following histopathological diagnosis with NASH. Intense pSmad3L immunostaining was present in hepatocytic nuclei throughout the liver lobules, while C-terminal phosphorylation of Smad3 was strongly suppressed in the nuclei of all hepatocytes (Figure 2B).

A severely fibrotic liver specimen with necroinflammatory grade 2 and fibrosis stage 4 obtained from patient 26 in Table 1, who was diagnosed with HCC at time of liver biopsy, showed high phosphorylation of Smad3L and suppressed C-terminal phosphorylation of Smad3 in hepatocytic nuclei (Figure 2C). Another severely fibrotic liver specimen with similar necroinflammatory activity, obtained from patient 17 in Table 1 who has not developed HCC during 15 years following liver biopsy, showed high phosphorylation in hepatocytic nuclei at Smad3C, but low phosphorylation at Smad3L (Figure 2D).

### 2.2. Although the pSmad3L Pathway in Hepatocytes Tended to Predominate While the pSmad3C Pathway Became Quiescent during Chronic Liver Disease Progression in both HCV and NASH, Smad3 Phospho-Isoforms in NASH Livers Varied more Widely than in HCV-Infected Livers at All Fibrotic Stages

**Figure 3:**
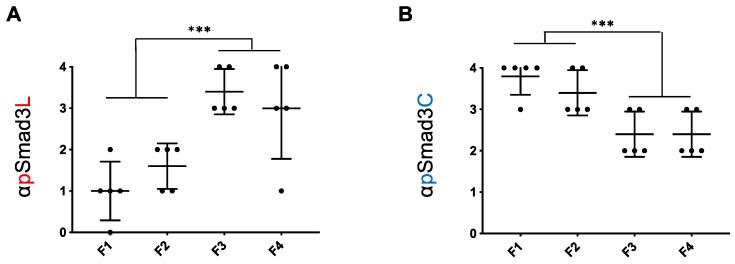
Hepatocytic Smad phospho-isoform signaling shifted from the pSmad3C pathway to the pSmad3L pathway as HCV-related chronic liver disorders progressed. (**A**) Extent of hepatocytic phosphorylation at Smad3L increased in relation to increasing fibrosis in HCV-related chronic liver disorders. Hepatocytic phosphorylation of Smad3L in highly fibrotic livers (fibrosis stage3 to 4) was significantly greater than in livers with stage1 to 2 fibrosis. *** *p* < 0.001 (**B**) Extent of phosphorylation at Smad3C decreased as fibrosis increased in HCV-related chronic liver disorders. Phosphorylation of Smad3C in hepatocytes of highly fibrotic livers (stage3 to 4) was lower than in livers with stage1 to 2 fibrosis. *** *p* < 0.001 Specimens from livers chronically infected with HCV were graded based on degree of fibrosis (1 to 4). Smad3 phosphorylation was scored from 0 to 4 as described in Methods. Dots (●) represent pSmad3L (**A**) and pSmad3C (**B**) positivity for each liver specimen. Horizontal bars indicate mean phospho-Smad3 positivity in each group. The linker region of Smad3 displayed very little phosphorylation in normal liver.

As HCV-infected livers progressed from chronic hepatitis to cirrhosis, HCC occurrence increase [34]. Cirrhosis is a prerequisite for development of HCV-related HCC, which occurs at a yearly rate of 4% [39]. Chronic inflammation from HCV infection simultaneously induced hepatic fibrosis and carcinogenesis, in highly fibrotic livers (stage 3 to 4), phosphorylation at Smad3L was significantly greater than in livers with mild fibrosis (stage1 to 2; Figure 3A). In contrast, Smad3C showed less phosphorylation in highly fibrotic livers than that in livers with stage 1 to 2 fibrosis (Figure 3B). These results are consistent with our previous finding that chronic inflammation associated with HCV infection shifts hepatocytic TGF-β signaling from tumor suppression to fibro-carcinogenesis, promoting liver fibrosis and carcinogenesis [34].

**Figure 4:**
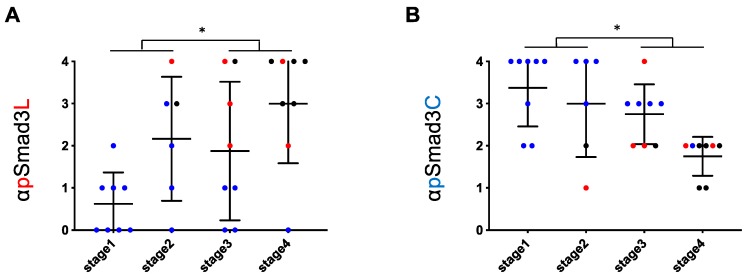
Although hepatocytic Smad phospho-isoform signaling shifted from the pSmad3C pathway to the pSmad3L pathway during chronic liver disease progression in NASH, Smad3 phospho-isoforms of NASH livers varied more widely than those of HCV-infected livers throughout all fibrotic stages. (**A**) Extent of hepatocytic phosphorylation at Smad3L tended to increase in proportion to fibrotic stage in NASH livers. Phosphorylation of Smad3L in the hepatocytes of highly fibrotic livers (F3 to F4) was greater than in livers with grade F1 to 2 fibrosis. (**B**) Extent of hepatocytic phosphorylation at Smad3C tended to decrease in proportion to fibrotic stage in NASH livers. Phosphorylation of Smad3C in the hepatocytes of highly fibrotic livers (F3 to F4) was lower than those in livers with grade F1 to 2 fibrosis. * *p* < 0.05 NASH livers were divided into four fibrotic stages. Smad3 phosphorylation in NASH livers was scored from 0 to 4 as described in Methods. Dods (●) represent pSmad3L (**A**) and pSmad3C (**B**) positivity for each liver specimen. Blue, red and black colors indicate hepatocytes from which HCC had not developed, HCC developed during the following period, and HCC already existed at liver biopsy, respectively. Horizontal bars indicate mean phospho-Smad3 positivity in each group.

As in HCV-infected livers, phosphorylation at Smad3L in highly fibrotic NASH livers (stages 3 and 4) was also significantly greater than in mildly fibrotic NASH livers (stages 1 and 2; Figure 4A). In contrast, phosphorylation at Smad3C in highly fibrotic NASH livers (stages 3 and 4) was less than in livers with stage 1 and 2 fibrosis (Figure 4B). In NASH livers, however, extent of phosphorylation at either Smad3L or Smad3C varied more widely than in HCV-infected livers throughout all fibrotic stages. Three of fourteen patients with NASH at fibrosis stages 1 and 2 fibrosis, but no patients with HCV-infected livers showing comparably mild fibrosis, had abundant Smad3L phosphorylation (scores 3 to 4; 21.4% vs. 0%, Figure 3A and Figure 4A). In contrast, 7 of 16 patients with NASH at fibrosis stages 3 and 4, but only 1 of 10 patients with HCV-infected livers with equally severe fibrosis, showed limited Smad3L phosphorylation (scores 0 to 2; 43.8% vs. 10%, Figure 3A and Figure 4A). Moreover, 4 of 14 patients with NASH and stage 1 or 2 fibrosis, but no patients with HCV infection and comparably mild fibrosis, showed limited Smad3C phosphorylation (scores 0 to 2; 28.6% vs. 0%, Figure 3A and Figure 4A).

### 2.3. Regardless of Fibrotic Stage, HCC Developed from Hepatocytes in NASH Livers with Abundant Phosphorylation at Smad3L and Little Phosphorylation at Smad3C

**Figure 5:**
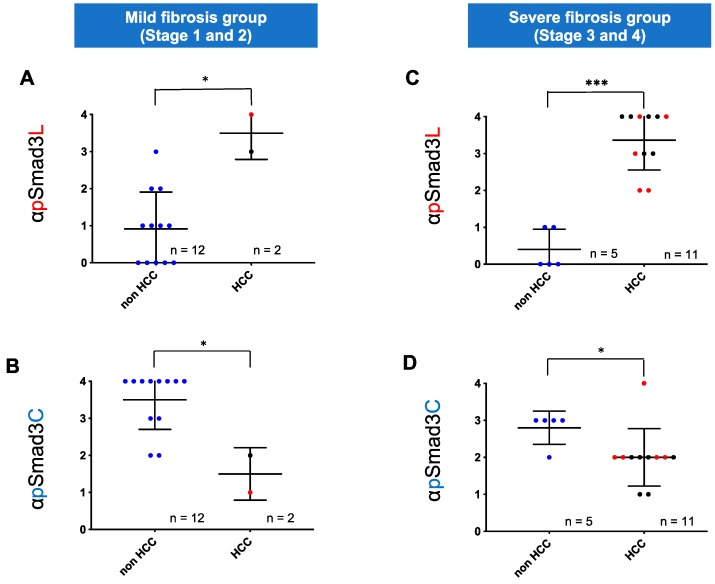
Regardless of fibrotic stage, HCC developed from hepatocytes with abundant phosphorylation at Smad3L and little phosphorylation at Smad3C in NASH livers. HCC developed from hepatocytes with abundant Smad3L phosphorylation (**A**) and limited Smad3C phosphorylation (**B**) in mildly fibrotic NASH livers (F1 to F2). In these livers, extent of phosphorylation at Smad3L (**A**) or Smad3C (**B**) in hepatocytes from which HCC developed was respectively greater or less than in those from which HCC did not arise (* *p* < 0.05). HCC did not develop from hepatocytes with limited Smad3L phosphorylation (**C**) and abundant Smad3C phosphorylation (**D**) even in severely fibrotic NASH livers. In severe fibrotic NASH livers (F3 to F4), the extent of phosphorylation at Smad3L (**C**) or Smad3C (**D**) in the hepatocytes from which HCC developed was respectively less or greater than those from which HCC did not arise (* *p* < 0.05 ***, *p* < 0.001). NASH liver fibrosis cases were classified as mild (F1 to 2) or severe (F3 to 4). Smad3 phosphorylation in NASH livers was scored from 0 to 4 as described in the Methods. Dots (●) represent pSmad3L (**A**,**C**) and pSmad3C (**B**,**D**) positivity for each liver specimen. Blue, red and black colors respectively indicate hepatocytes from which HCC did not develop, HCC developed during follow-up, and HCC already was evident at time of liver biopsy. Horizontal bars indicate mean phospho-Smad3 positivity in each group.

Two of three patients with NASH livers showing mild fibrosis and abundant Smad3L phosphorylation developed HCC during follow-up (Figure 4A), while 5 of 7 patients with NASH livers showing severe fibrosis and limited Smad3L phosphorylation did not develop HCC (Figure 4A). In contrast, 2 of 4 patients with NASH livers showing mild fibrosis and limited Smad3C phosphorylation developed HCC (Figure 4B). Accordingly, we divided NASH liver fibrosis into mild and severe fibrotic stages, and further investigated correlation of hepatocytic pSmad3L/C positivity with HCC occurrence (Figure 5). Two of fourteen patients with mild fibrosis (stages 1 and 2) were found to have HCC at time of biopsy or during follow-up; their hepatocytic nuclei showed abundant Smad3L phosphorylation (scores 3 to 4) and little Smad3C phosphorylation (scores 1 to 2; Figure 5A,B). On the other hand, 5 of 16 patients with severe fibrosis (stages 3 and 4) did not develop HCC during follow-up; they showed limited Smad3L phosphorylation (scores 0 to 1) and abundant Smad3C phosphorylation (score, 3; Figure 5C,D). Hepatocytes strongly positive for pSmad3L, therefore, can develop HCC even in the setting of mild fibrosis, while hepatocytes positive for pSmad3C did not develop HCC despite advanced fibrosis.

### 2.4. NASH Patients with Hepatocytes Positive for pSmad3L and Negative for pSmad3C Increased Risk of HCC Development

**Figure 6:**
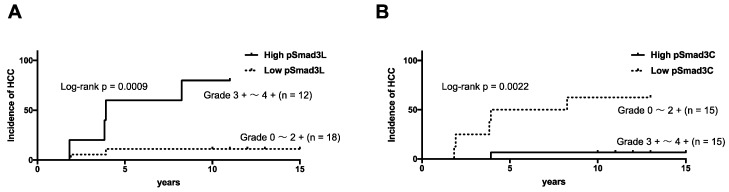
NASH patients whose hepatocytes were strongly positive for pSmad3L and essentially negative for pSmad3C were more likely to develop HCC. (**A**) HCC was likely to occur among patients with NASH specimens strongly positive for pSmad3L. HCC also was significantly more likely in patients with abundant Smad3L phosphorylation (scores 3 to 4, solid line) than in those with sparse Smad3L phosphorylation (scores 0 to 2, dotted line). (**B**) HCC did not occur among patients with hepatocytes in NASH specimens strongly positive for pSmad3C. HCC incidence also was significantly higher in patients with sparse Smad3C phosphorylation (scores 0 to 2; dotted line) than in those with abundant Smad3C phosphorylation (scores 3 to 4; solid line). Occurrence rates were compared using Kaplan–Meier analysis and log-rank tests.

We then investigated whether Smad3 phosphorylation levels could affect the risk of neoplastic evolution in patients with NASH. To compare HCC incidence, patients were classified into those with abundant (scores 3 to 4) and limited (scores 0 to 2) Smad3 phosphorylation in hepatocytic nuclei. HCC developed in 11 of 12 patients with abundant Smad3L phosphorylation, but in only 2 of 18 patients in limited Smad3L phosphorylation (log-rank 0.0009; Figure 6A). In contrast, HCC developed in 12 of 15 patients with limited Smad3C phosphorylation, but in only 1 of 15 patients with abundant Smad3C phosphorylation, even after more than 10 years (log-rank 0.0022; Figure 6B).

We finally examined whether abundant Smad3L phosphorylation and limited Smad3C phosphorylation in hepatocytes were independent predictive factors for future HCC onset. We performed statistical analysis for pSmad3C and pSmad3L positivity, necroinflammatory grade, and for fibrosis stage in patients without HCC at the time of liver biopsy. In univariate analysis, abundant phosphorylation at Smad3L, limited phosphorylation at Smad3C, and a high fibrosis stage showed P values less than 0.05, and these 3 variables were subjected to multivariate analysis, in which all were shown to be independent predictive factors of HCC development within 10 or more years (Table 3).

Collectively, HCC rarely developed in patients with strong phosphorylation at Smad3C, while HCC often observed in patients with strong phosphorylation at Smad3L in hepatocytic nuclei of NASH livers.

## 3. Discussion

Our current studies describe how Smad phospho-isoform signaling is similar but yet different between NASH- and HCV-related chronic liver diseases. As disease progress from chronic hepatitis to cirrhosis in patients with NASH- and HCV-related disease related pSmad3L pathway in hepatocytes tended to predominate, on the other hand, pSmad3C pathway decreased. Interestingly, NASH patients whose livers showed pSmad3L dominance in hepatocytic nuclei develop HCC in near future even in scarce fibrotic livers. Alternatively, pSmad3C dominant livers did not develop HCC even in cirrhotic livers. These data suggest that Smad phospho-isoforms may be important biomarkers for prediction of HCC in NASH, and also may represent a potentially therapeutic target for prevention of NASH-related HCC.

In the damaged hepatocytes of patients with either NASH or HCV infection, Smad phospho-isoform signaling tends to shift from the tumor-suppressive pSmad3C pathway to the oncogenic pSmad3L pathway. In NASH-rerated cirrhosis, however, the yearly cumulative incidence of HCC was 2.6%, which is lower than in HCV-related cirrhosis [39]. On the other hand, NASH-related HCC sometimes occurred even in mildly fibrotic livers [40]. Notably, progression to HCC in NASH was much less predictable than in HCV-related liver disease, where progression is tightly linked to hepatitis severity, and effective treatment achieving sufficient viral elimination can halt progression of fibrosis. Fibrosis in NASH can regress spontaneously in early stages, while NASH livers with early fibrosis still can develop HCC [41]. Yet, even cirrhotic livers in NASH showed less HCC development than in HCV-related cirrhosis [39]. Underlying these differences, NASH- and HCV-infected livers exhibited notably different Smad phospho-isoform signaling patterns, with NASH being more variable.

Fibrosis in NASH can progress to cirrhosis for many years without HCC development. Hepatic fibrosis is characterized by accumulation of ECM proteins, regardless of underlying etiology. Matrix deposition is dynamic and involves phases of both progression and regression during pathologic processes [42]. When synthesis of ECM exceeds degradation, accumulation of ECM leads to liver fibrosis. Strong expression of plasminogen activator inhibitor-1 (PAI-1), the major physiologic inhibitor of plasminogen activator, reduces extracellular matrix degradation by perturbing the plasminogen activation system [43,44]. As a result of chronic liver damage, hepatic stellate cells (HSC), the principal effectors of ECM deposition, undergo progressive activation to become myofibroblast (MFB)-like cells [45]. MFB usually retain fibrogenic TGF-β signaling while losing capacity to respond to cytostatic TGF-β signaling [18]. This molecular shift is suggested by differential cellular localization of pSmad2L and pSmad3L observed in MFB. Activated by pro-inflammatory cytokines, JNK stimulates nuclear accumulation of pSmad3L in MFB, while inhibiting cytostatic pSmad3C signaling [34,46,47]. On the other hand, Smad2, which resembles Smad3 but has several distinguishing features, can accumulate in the nucleus only if its C-terminus is phosphorylated under conditions of sustained linker phosphorylation by JNK [48]. Translocation of both linker and C-terminally phosphorylated Smad2 (pSmad2L/C) to the nucleus cooperates with pSmad3L and Smad4 to enhance PAI-1 transcription and promote hepatic fibrosis [38]. As a result, MFB lose growth-inhibitory responsiveness to TGF-β while TGF-β continues to accelerate ECM accumulation. Both HCV-infected liver and NASH liver specimens display pSmad3L signal in nuclei of α-smooth muscle actin-immunoreactive MFB in the portal tracts, leading to liver cirrhosis in both diseases. Similarly to MFB, hepatocytes in cirrhotic livers infected with HCV exhibit strong phosphorylation at Smad3L. In contrast, degree of linker phosphorylation of Smad3 in hepatocytic nuclei remained low (staining scored as 0 or 1) in all patients with advanced NASH-related fibrosis who did not develop HCC. 

NASH can regress without specific pharmacologic intervention; for example, weight loss through lifestyle modification improved NAFLD activity scores [49,50]. On the other hand, chronic inflammation induced by persistent HCV infection drives a maladaptive reparative reaction and over stimulates liver cell death and regeneration, promoting liver fibrosis and carcinogenesis [51]. Degree of fibrosis correlates closely with risk of HCC in HCV infected livers. In contrast, HCC occasionally develops in relatively mildly fibrotic NASH livers [40]. These results indicate differential mechanisms of NASH- and HCV-associated carcinogenesis, especially in early stages of chronic hepatitis. Because extent of Smad3L phosphorylation increases as fibrotic stage progresses in chronic hepatitis C, Smad3L showed little phosphorylation in early chronic hepatitis C. In contrast, degree of linker phosphorylation of Smad3 in hepatocytic nuclei was high (staining scored as 3 or 4) in all patients with mildly fibrotic NASH livers (F1 to F2) that developed HCC.

**Figure 7:**
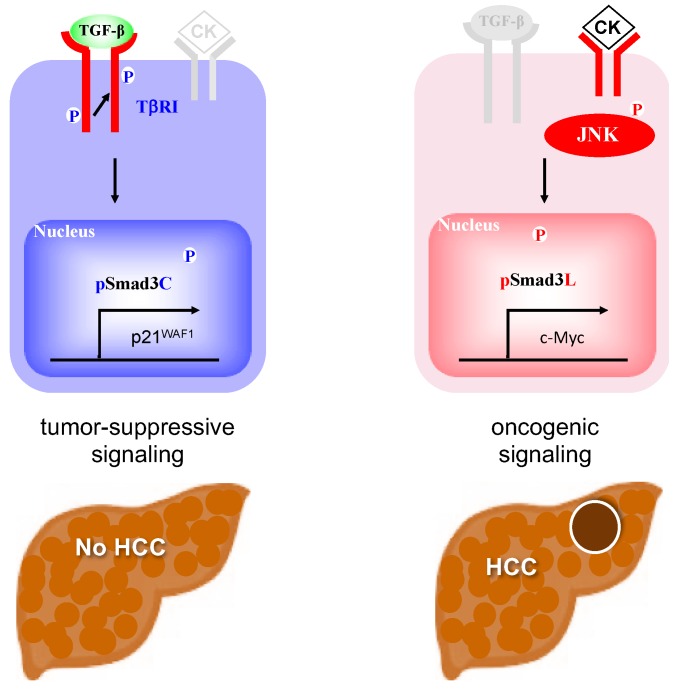
Pro-inflamatory cytokine (CK) such as tumor necrosis factor (TNF)-α and interleukin 6 (IL-6) shifted hepatic TGF-β signaling from the tumor-suppressive pSmad3C pathway to the oncogenic JNK-dependent pSmad3L pathway in NASH liver.

Obesity-associated insulin resistance induces abnormal production of inflammatory cytokines such as tumor necrosis factor (TNF)-α and interleukin 6 (IL-6). These inflammatory cytokines activate the JNK pathway promoting hepatocyte proliferation [52,53]. During carcinogenesis, the physiologic balance between proliferation and differentiation in normal hepatocyte homeostasis is lost. Cytostatic TGF-β/pSmad3C signaling appears to maintain such balance in early stages of carcinogenesis. JNK acts as an important regulator of Smad3 signaling by increasing the basal amount of hepatocytic pSmad3L available for cell growth, while limiting the cytostatic action of pSmad3C [54,55,56]. However, abundant phosphorylation at Smad3L and limited phosphorylation at Smad3C in NASH livers may be important risk factors for and drivers of HCC development (Figure 7). Smad phospho-isoforms should serve as important new biomarkers for HCC risk assessment in patients with NASH.

## 4. Materials and Methods

### 4.1. Patient Enrollment and Clinical/Biochemical Evaluation

From January 2003 to December 2007 at the Department of Gastroenterology and Hepatology of Kansai Medical University Hospital, 100 patients were diagnosed histologically with NASH based on liver biopsy findings in the absence of other liver diseases such as viral hepatitis, alcoholic hepatitis, autoimmune hepatitis, and drug-induced liver disease. Among patients diagnosed with NASH, 30 could be followed up continuously for 10 years or more until onset of HCC. Ultrasound (US) or computed tomography (CT) was performed for every 6 months to detect HCC. Laboratory tests included blood cell counts and measurements of serum concentrations of aspartate aminotransferase (AST), alanine aminotransferase (ALT), total bilirubin, albumin, and prothrombin, using standard clinical laboratory techniques. Body mass index (BMI) was calculated as weight (kg)/[height (m)]^2^, with obesity defined as BMI exceeding 25 kg/m^2^. The Fib-4 index was calculated as [age (yr) × AST (IU/L)]/[(PLT (x10^9^/L) × √ALT (IU/L)]. We also studied 20 randomly chosen patients at each stage of HCV-related fibrotic liver disease (F1–F4) as controls. Patients were enrolled in the study after informed consent and following the approval and recommendations of the Ethics Review Board of Kansai Medical University (code: 2019067).

### 4.2. Evaluation of Liver Biopsy Specimens

All enrolled patients underwent percutaneous liver biopsy under US guidance. The liver specimens were embedded in paraffin and stained with hematoxylin-eosin. The specimens were evaluated by two hepatopathologists (K.T. and M.I.) who were blinded to clinical findings. Histologic grading and staging used the Brunt Criteria, an internationally accepted standard in pathologic practice [57].

### 4.3. Domain-Specific Antibodies (Abs) against Phosphorylated Smad2 and Smad3

Polyclonal anti-phospho-Smad3 [anti-pSmad3L (Ser. 208/213) and anti-pSmad3C (Ser. 423/425)] respectively were raised against phosphorylated linker and C-terminal regions of Smad3 by immunizing rabbits with synthetic peptides. Relevant antisera were affinity-purified using phosphorylated peptides. We confirmed that these domain-specific Abs selectively distinguished between phosphorylated linker regions and phosphorylated C-terminal regions of Smad3, as described previously [38].

### 4.4. Immunohistochemistry

Immunohistochemical analyses were performed as described previously. Primary Abs used in this study included rabbit polyclonal anti-pSmad3L (2 μg/mL) and rabbit polyclonal anti-pSmad3C (0.5 μg/mL) as described above.

For immunohistochemical analyses, sections exposed to primary Abs then were incubated with peroxidase-labeled polymer conjugated to anti-rabbit immunoglobulin G (IgG, DAKO, Santa Clara, CA, USA). Finally, staining was developed with 3,3′-diaminobenzidine tetrahydrochloride (DAB; Vector Laboratories, Burlingame, CA), counterstained with Mayer’s hematoxylin (Merck, Darmstadt, Germany), and mounted under coverslips. We counted and scored pSmad3C/L positivity in the nuclei of hepatocytes. Hepatocytic Smad3 phosphorylation was scored as follows; 0, no positivity; 1, <25% Smad3 phosphorylation; 2, 25% to 50% Smad3 phosphorylation; 3, 50% to 75% Smad3 phosphorylation; and 4, >75% Smad3 phosphorylation, as described previously [34].

### 4.5. Statistical Analysis

HCC incidence during the follow-up period after liver biopsy was determined using the Kaplan–Meier method. HCC occurrence curves were compared between abundant (score 3–4) and sparse (score 0–2) Smad3L/C phosphorylation by means of the log-rank test. For continuous variables, the optimal cutoff threshold for defining the group was set using receiver operating characteristic curves. All parameters with a P value less than 0.10 in univariate analysis were subjected to multivariate analysis using a Cox proportional hazards model, and those with P values below 0.05 were considered significant. The Mann-Whitney U test was used to identify significant differences between hepatocytic pSmad3L and pSmad3C positivity according to fibrosis stage, with or without HCC occurrence.

## 5. Conclusions

During progression of NASH-related liver disease, hepatocytic Smad phospho-isoforms come to reflect liver fibrosis progression and carcinogenesis, shifting from tumor-suppressive pSmad3C to carcinogenic pSmad3L. HCC was rarely developed in NASH livers with severe fibrosis but limited Smad3L phosphorylation. On the other hand, the risk of HCC was likely to increase in those with mild fibrosis but limited Smad3C phosphorylation. Thus, abundant phosphorylation at Smad3L and limited phosphorylation at Smad3C may be important risk factors for HCC in NASH. Smad phospho-isoforms may serve as important new biomarkers for HCC risk assessment in NASH.

## Figures and Tables

**Table 1:** Clinicopathologic features and Smad2/3/L/C phosphorylation in liver specimens from patients with NASH.

	No.	Age	Sex	AST	ALT	PLT	FiB4	T-Bil	ALB	PT	F Stage	NI Grade	pSmad3L Staining	pSmad3C Staining	Folluw-up Period	Time to HCC *
No HCC	1	27	M	145	319	25.1	0.87	0.9	5.1	118	1	1	1	4	12	
	2	29	F	150	248	28.0	0.99	0.7	4.3	75	1	1	1	4	12	
	3	38	M	59	178	21.1	0.8	0.8	4.9	101	1	1	0	4	10	
	4	60	F	90	118	26.3	1.89	0.5	4.0	102	1	1	1	3	11	
	5	63	F	38	43	28.4	1.29	0.5	4.9	110	1	1	0	4	15	
	6	68	F	84	82	28.3	2.23	1.1	4.4		1	1	0	2	13	
	7	72	F	49	45	20.1	2.62	0.6	4.2	127	1	1	0	2	13	
	8	70	M	168	214	16.9	4.76	1.2	4.4	74	1	2	2	4	11	
	9	44	M	93	169	23.9	1.32	1.4	4.5	118	2	1	2	4	13	
	10	45	F	84	145	23.1	1.36	0.7	4.9	119	2	1	1	3	10	
	11	66	F	29	29	19.2	1.85	0.5	4.2	115	2	1	0	4	13	
	12	65	M	98	35	19.3	5.58	0.4	4	88	2	2	3	4	11	
	13	46	M	27	14	11.3	2.94	0.8	3.9	88	3	1	1	3	11	
	14	54	M	40	86	21	1.11	1.1	4.4	89	3	2	0	3	11	
	15	56	F	155	192	12.5	5.01	1.1	4.3		3	2	1	3	12	
	16	57	F	30	24	16.9	2.07	0.7	4.3	107	3	2	0	3	10	
	17	54	F	80	99	12.8	3.39	1.2	4.4	101	4	2	0	2	13	
HCC	18	50	M	51	70	9.1	3.35	1.2	4	38	2	1	4	1		8.25
	19	60	M	19	14	17.3	1.76	0.4	4.7	126	2	1	3	2		0
	20	72	M		53	9.8	6.06	0.8	3.8	84	3	1	3	4		3.92
	21	75	M	44	38	15.8	3.39	0.9	4	92	3	1	4	2		0
	22	78	F	31	21	4.3	12.27	1.7	3.3	64	3	1	2	2		1.92
	23	69	M	50	39	9.1	6.07	1.3	3.8	83	3	2	4	2		1.83
	24	58	M	64	61	5	9.51	2.2	4	67	4	1	3	2		0
	25	73	M	64	72	8.4	6.55	1	3.1	44	4	1	3	1		0
	26	71	M	129	73	9.9	10.83	2.9	3.7	72	4	2	4	1		0
	27	76	F	59	29	8.4	9.91	1.2	3.2	77	4	2	2	2		3.92
	28	80	F	28	16	4.4	12.73	1.2	3.1	64	4	2	4	2		0
	29	69	F	48	23	7	9.87	2.4	2.6	50	4	3	4	2		3.83
	30	75	M	33	14	10.4	6.36	0.9	3.6	87	4	3	4	2		0

* 0 indicates that HCC had already occurred when liver biopsy was performed. NASH, nonalcoholic steatohepatitis. HCC, hepatocellular carcinoma. F, fibrosis. NI, necroinflammatory.

**Table 2:** Clinicopathologic features and Smad3L/C phosphorylation in liver specimens from patients with hepatitis C virus (HCV).

No.	Age	Sex	AST	ALT	PLT	FiB4	T-Bil	ALB	PT	F Stage	NI Grade	pSmad3L Staining	pSmad3C Staining
1	32	F	18	21	17.8	0.71	0.7	4.2	100	1	1	0	4
2	44	M	244	156	16.5	5.21	0.9	5.1	88	1	2	1	4
3	63	F	27	24	31.4	1.11	0.5	4.7	110	1	1	1	3
4	45	M	81	65	17.7	2.55	0.6	0.7	104	1	2	2	4
5	51	M	29	34	13.3	1.91	0.7	4.3	92	1	2	1	4
6	70	F	22	31	13.9	1.99	0.6	3.9	105	2	2	1	4
7	45	M	57	101	12.7	2.01	0.7	4.4	88	2	3	2	3
8	72	F	297	197	11.6	13.13	1.2	3.8	102	2	2	2	4
9	64	M	105	83	16.8	4.39	0.7	4.1	77	2	2	1	3
10	69	F	94	75	23.4	3.2	1.2	3.9	81	2	2	2	3
11	57	M	41	43	15.0	2.38	0.7	4.6	107	3	2	3	3
12	59	M	161	169	11.4	6.41	1.0	4.2	89	3	2	4	2
13	68	M	83	93	7.8	7.5	1.7	3.4	71	3	3	3	2
14	55	M	30	26	15.7	2.06	1.2	3.9	72	3	2	3	2
15	64	F	43	31	14.4	3.43	1.1	4.5	63	3	2	4	3
16	69	F	26	19	13.7	3.00	1.2	4.7	88	4	2	4	2
17	58	M	64	69	11.0	4.06	1.2	4.2	96	4	3	4	3
18	48	M	62	103	13.2	2.22	0.6	4.3	108	4	2	1	2
19	34	F	96	79	15.3	2.40	1.0	4.1	83	4	2	3	3
20	61	F	87	56	9.0	7.88	0.4	3.9	79	4	1	3	2

HCV, hepatitis C virus. F, fibrosis. NI, necroinflammatory.

**Table 3:** Phospho-Smad3L/C and fibrosis stage are independent predictors of HCC occurrence.

Variables	Category	Univariate Analysis		Multivariate Analysis	
		Hazard Ratio (95% CI)	*p* Value	Hazard Ratio (95% CI)	*p* Value
Fibrosis stage	Low (1 and 2)				
	High (3 and 4)	8.823 (1.023–76.12)	0.04766	22.36 (1.269–394.1)	0.0338
Necroinflammatory grade	Low (0 and 1)				
	High (2 and 3)	1.768 (0.3563–8.771)	0.4857		
NAFLD activity score	<5				
	≥5	0	0.999		
pSmad3C positivity	High (3 and 4)				
	Low (1 and 2)	13.25 (1.538–114.2)	0.01868	15.61 (1.254–194.3)	0.0327
pSmad3L positivity	Low (1 and 2)				
	High (3 and 4)	10.32 (1.866–57.05)	0.007479	18.53 (1.712–200.6)	0.0163
